# Overexpression of long non-coding RNA AP001505.9 inhibits human hyaline chondrocyte dedifferentiation

**DOI:** 10.18632/aging.202833

**Published:** 2021-04-04

**Authors:** Lin Chen, Jinying Xu, Shuang Lv, Yan Zhao, Dongjie Sun, Yangyang Zheng, Xianglan Li, Lihong Zhang, Guangfan Chi, Yulin Li

**Affiliations:** 1 The Key Laboratory of Pathobiology, Ministry of Education, College of Basic Medical Sciences, Jilin University, Changchun, Jilin, China; 2 Department of Gastrointestinal and Colorectal Surgery, China-Japan Union Hospital of Jilin University, Changchun, Jilin, China; 3 Department of Operating Room, China-Japan Union Hospital of Jilin University, Changchun, Jilin, China; 4 Department of Dermatology, China-Japan Union Hospital of Jilin University, Changchun, Jilin, China

**Keywords:** long non-coding RNA AP001505.9, dedifferentiation, hyaline chondrocyte, autologous chondrocyte implantation, osteoarthritis

## Abstract

Autologous chondrocyte implantation (ACI) is an effective method for treating chronic articular cartilage injury and degeneration; however, it requires large numbers of hyaline chondrocytes, and human hyaline chondrocytes often undergo dedifferentiation *in vitro*. Moreover, although long non-coding RNAs (lncRNAs) regulate gene expression in many pathological and physiological processes, their role in human hyaline chondrocyte dedifferentiation remains unclear. Here, we examined lncRNA and mRNA expression profiles in human hyaline chondrocyte dedifferentiation using microarray analysis. Among the many lncRNAs and mRNAs that showed differential expression, lncRNA AP001505.9 (ENST00000569966) was significantly downregulated in chondrocytes after dedifferentiation. We next performed gene ontology, pathway, and CNC (coding-non-coding gene co-expression) analyses to investigate potential regulatory mechanisms for AP001505.9. Pellet cultures were then used to redifferentiate dedifferentiated chondrocytes, and AP001505.9 expression was upregulated after redifferentiation. Finally, both *in vitro* and *in vivo* experiments demonstrated that AP001505.9 overexpression inhibited dedifferentiation of chondrocytes. This study characterizes lncRNA expression profiles in human hyaline chondrocyte dedifferentiation, thereby identifying new potential mechanisms of chondrocyte dedifferentiation worthy of further investigation.

## INTRODUCTION

Articular cartilage injury and degeneration are among the most common diseases worldwide, and their prevalence increases with patient age [1, 2]. The lack of blood vessels and nerves within articular cartilage forces it to rely on diffusion of nutrients from the synovial fluid. Self-repair following injury can therefore prove challenging, and osteoarthritis (OA) may occur if the cartilage is not adequately repaired. Typical pathological features of OA include chondrocyte degeneration, extracellular matrix degradation, and osteogenesis [3]. OA can occur in multiple joints and causes clinical symptoms such as joint pain, swelling, stiffness, deformity, and limited movement [4–6]. Although symptoms can be relieved by medication in the early stages of disease [7, 8], patients may eventually require surgical interventions, including artificial joint replacement [9]. Although novel and effective treatment options, including microfracture and autologous cartilage grafts, have been developed for cartilage injuries, challenges remain, including limited cartilage sources, small repair areas, and fibrocartilage repair after microfracture [10, 11].

Autologous chondrocyte implantation (ACI) is an effective method for the treatment of chronic articular cartilage defects. ACI technology was first introduced in 1994 and is currently in its third generation, which is known as matrix-associated ACI (MACI) [12]. ACI technology involves obtaining a small piece of tissue with autologous hyaline chondrocytes from a healthy site, isolating and subculturing hyaline chondrocytes *in vitro*, and implanting the cells at the site of cartilage defect, thus repairing the defect [13]. However, human hyaline chondrocytes often undergo dedifferentiation *in vitro* [14]. Dedifferentiation is a process in which well-differentiated mature cells gradually lose their differentiated phenotype and transform into undifferentiated cells [15]. Dedifferentiation of human hyaline chondrocytes occurs during culture passaging *in vitro* and results in downregulation of the expression of hyaline chondrocyte marker genes, such as *COL2* (type II collagen) and *SOX*-9 (SRY-Box transcription factor 9), and upregulation of fibrosis chondrocyte marker genes, such as *COL1* (type I collagen) [16, 17]. Dedifferentiation thus poses significant obstacles to obtaining sufficient numbers of hyaline chondrocytes for transplantation. Two different methods for redifferentiating chondrocytes have been developed to overcome this challenge. One method involves the addition of various cytokines, such as transforming growth factor (TGF)-β1, bone morphogenic protein (BMP)-2, and growth differentiation factor 5 (GDF-5) [18–20]. The other employs three-dimensional cultures, such as pellet, suspension, and gel cultures [21, 22]. Furthermore, studies have reported a “dedifferentiated-like” phenotype that might contribute to chondrocyte degeneration [23]. However, relatively little is known about the mechanisms underlying dedifferentiation, and additional research on these mechanisms is needed to facilitate the development of effective ACI technology [24].

Long non-coding RNAs (lncRNAs), which are non-coding RNA molecules more than 200 nucleotides in length, can be divided into five categories: sense lncRNA, anti-sense lncRNA, bidirectional lncRNA, intronic lncRNA, and intergenic lncRNA [25]. LncRNAs regulate gene expression in many biological processes, including cell proliferation, differentiation, and apoptosis [26–28]. Moreover, many lncRNAs that play roles in OA, such as lncRNA-HIT and ROCR, are present in cartilage [29]. Song et al. revealed that lncRNA-GAS5 contributes to the pathogenesis of OA by inhibiting microRNA-21 (miR-21) [30]. In addition, Su et al. reported that lncRNA-MEG3 inhibits angiogenesis in OA [31]. Moreover, lncRNAs can function as molecular sponges by binding to corresponding miRNAs and suppressing their ability to inhibit their target genes, thereby promoting gene expression [32]. Liu et al. reported that the TMSB4 pseudogene lncRNA promotes cartilage degeneration in human OA by acting as a competing endogenous RNA (ceRNA) [33]. However, the roles of lncRNAs in human hyaline chondrocyte dedifferentiation remain unknown.

In this study, we characterized lncRNA expression profiles during human hyaline chondrocyte dedifferentiation. Among them, we further investigated the mechanisms by which AP001505.9, a representative lncRNA, affected chondrocyte dedifferentiation, thereby laying a foundation for further study of the pathogenesis of chondrocyte dedifferentiation and OA.

## RESULTS

### Human hyaline chondrocytes undergo dedifferentiation during passage culture

**Figure 1:**
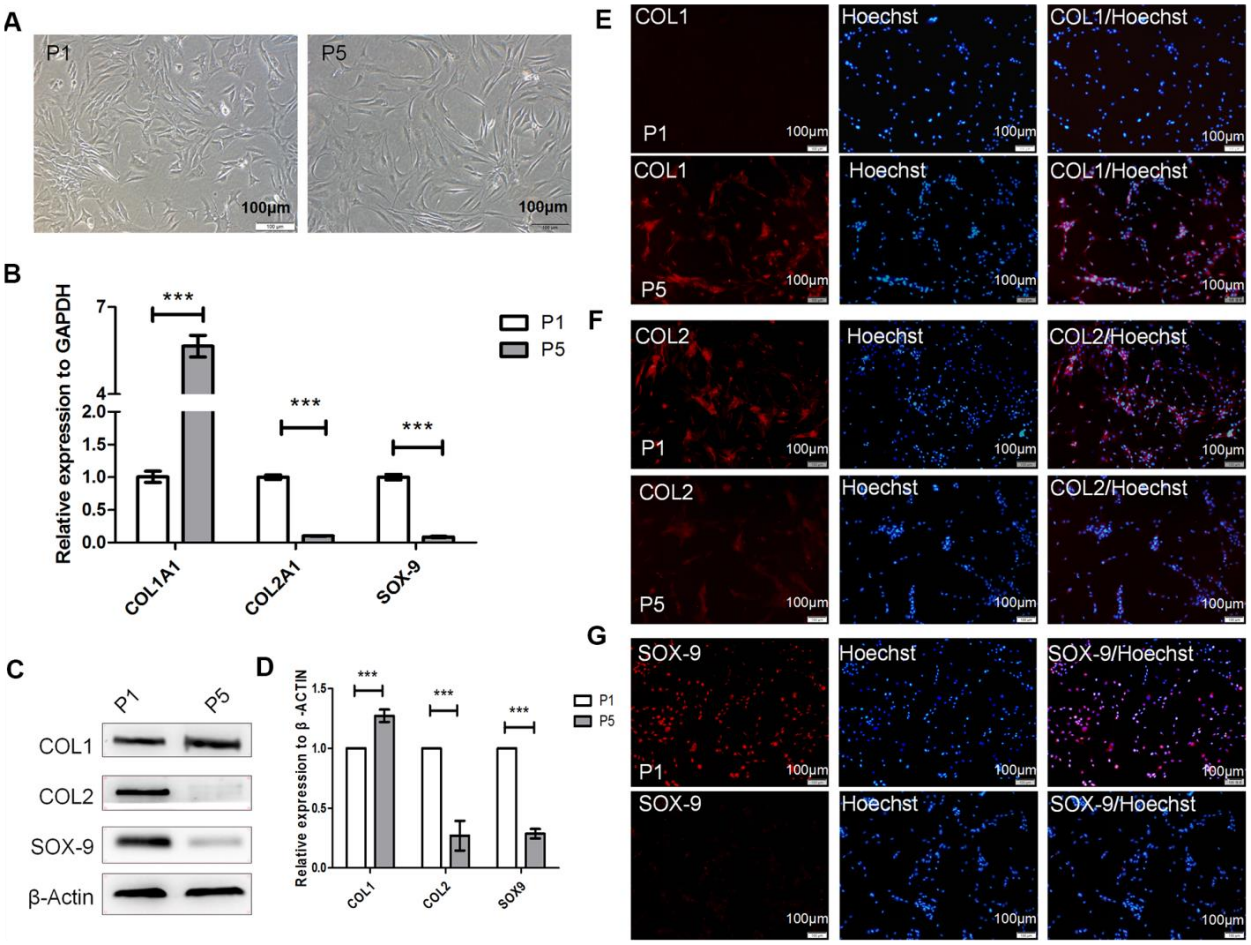
**Dedifferentiation of human hyaline chondrocytes occurred during passage culture *in vitro*.** (**A**) Morphological observations of passage 1 (P1) and P5 chondrocytes. (**B**) Real-time quantitative PCR (real-time qPCR) results for *COL1A1* (alpha-1 type I collagen), *COL2A1*, and *SOX9* expression in P1 and P5 chondrocytes. *GAPDH* (glyceraldehyde-3-phosphate dehydrogenase) was used as the internal reference. Data are represented as means ± standard deviation (n = 3). (**C**, **D**) Western blot analysis for COL1, COL2, and SOX-9 levels in P1 and P5 chondrocytes. β-actin was used as the internal reference. (**E**–**G**) Immunofluorescence staining of COL1, COL2, and SOX-9. P1 and P5 chondrocytes were stained with anti-COL2, anti-COL1, anti-SOX-9, and Hoechst 33342. *P < 0.05, **P < 0.01, ***P < 0.001. Scale bar, 100μm.

To confirm whether human hyaline chondrocytes undergo dedifferentiation *in vitro*, we collected and cultured primary chondrocytes from three donors. During passage, the shapes of the human hyaline chondrocytes gradually changed from polygonal, or near circular, to flat and long or spindly (Figure 1A). Compared with passage one (P1) chondrocytes, COL2A1 and SOX-9 expression was significantly downregulated (*P* < 0.001), while COL1A1 was upregulated (*P* < 0.001, Figure 1B), in P5 chondrocytes. Moreover, COL2 and SOX-9 levels were significantly lower in P5 than in P1 chondrocytes, while COL1 levels were significantly higher in P5 chondrocytes compared to P1 chondrocytes (*P* < 0.001, Figure 1C–1G). These results suggest that dedifferentiation of cultured human hyaline chondrocytes occurs during cell passage *in vitro*. P1 chondrocytes therefore served as differentiated chondrocytes, while P5 chondrocytes served as dedifferentiated chondrocytes, in subsequent experiments.

### lncRNA expression profiles in human chondrocyte dedifferentiation

**Figure 2:**
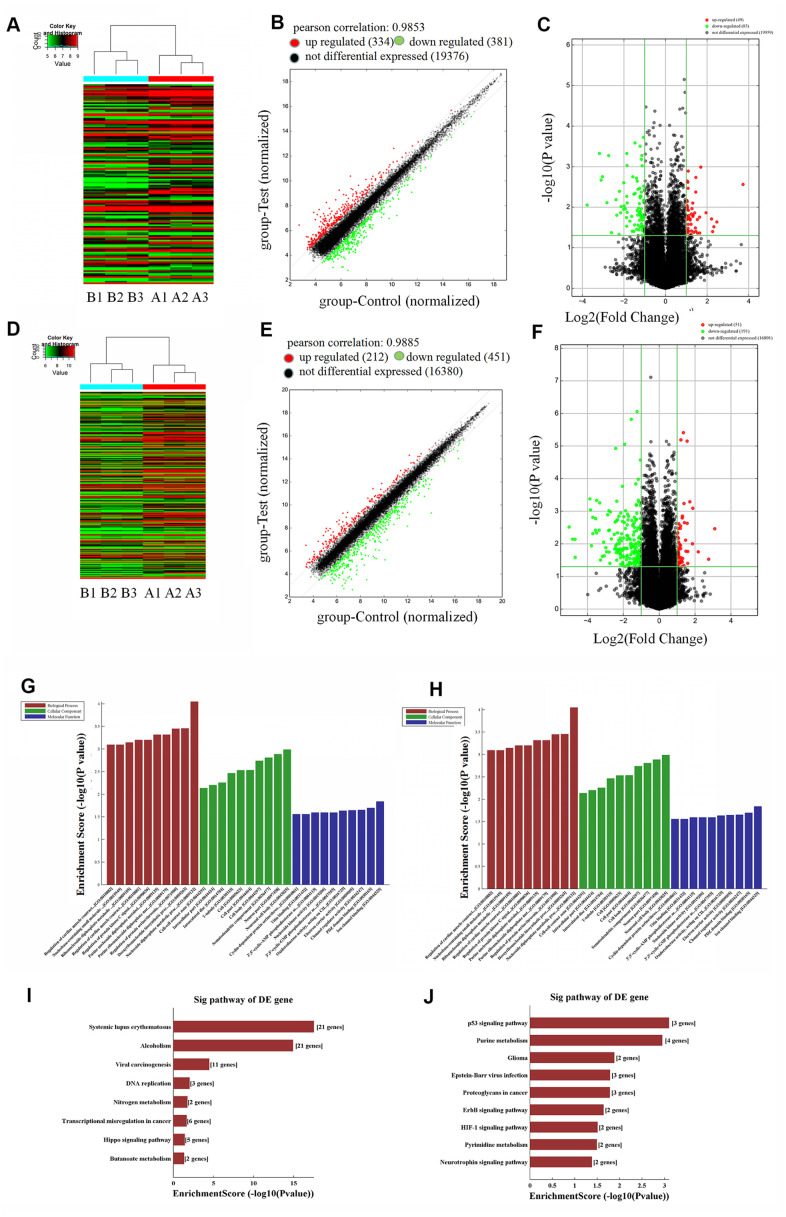
**Microarray analysis of lncRNAs (long non-coding RNA) and mRNAs in P1 and P5 chondrocytes (P1: A1, A2, A3; P5: B1, B2, B3).** (**A**) Thermal map of lncRNAs, y-axis: relative intensity of lncRNAs; (**B**) scatter plot of lncRNAs, Pearson correlation = 0.9853, upregulated (red) lncRNAs = 334, downregulated (green) lncRNAs = 381, not differentially expressed (black) lncRNAs = 19,376; (**C**) volcano plot of upregulated lncRNAs (red) = 49, downregulated (green) lncRNAs = 83, not differentially expressed (black) =19,959. (**D**) Thermal map of mRNAs, y-axis: relative intensity of mRNAs. (**E**) Scatter plot of mRNAs, Pearson correlation = 0.9885, upregulated (red) mRNAs = 212, downregulated (green) mRNAs = 451, not differentially expressed (black) mRNAs = 16,380. (**F**) volcano plot of mRNAs, upregulated (red) mRNAs = 51, downregulated (green) mRNAs = 191, not differentially expressed (black) mRNAs = 16,801. (**G**) Top ten downregulated biological processes, cellular components, and molecular functions in P5 chondrocytes compared to P1 cells. (**H**) Top ten upregulated biological processes, cellular components, and molecular functions in P5 chondrocytes. (**I**) Top eight downregulated pathways in P5 chondrocytes. (**J**) Top nine upregulated pathways in P5 chondrocytes.

To further explore the effects of lncRNAs in human hyaline chondrocyte dedifferentiation *in vitro*, microarray analysis of P1 and P5 human chondrocytes was performed. Compared to P1 human chondrocytes, 334 upregulated and 381 downregulated lncRNAs were identified in P5 chondrocytes. Forty-nine of the upregulated lncRNAs showed a greater than two-fold increase in expression (*P* < 0.05), while 83 of the downregulated lncRNAs showed a greater than two-fold decrease (*P* < 0.05), in P5 chondrocytes. Furthermore, seven lncRNAs showed a greater than five-fold upregulation, and 13 lncRNAs showed a greater than five-fold downregulation, in P5 cells (*P* < 0.05, Figure 2A–2C). The top 20 lncRNAs with the most significant differential expression are shown in Supplementary Table 1. These results indicate that expression of a large number of lncRNAs is altered during human chondrocyte dedifferentiation, suggesting that lncRNAs may play a regulatory role in this process *in vitro*; additionally, lncRNA downregulation occurs more often than upregulation during chondrocyte dedifferentiation.

We also identified mRNAs that are differentially expressed during the dedifferentiation process. Microarray analysis revealed that 212 mRNAs were upregulated and 451 were downregulated in P5 human chondrocytes compared to P1 chondrocytes. Moreover, expression of 51 of the upregulated mRNAs increased more than two-fold (*P* < 0.05), while expression of 191 of the downregulated mRNAs decreased by more than two-fold (*P* < 0.05), in P5 chondrocytes. Additionally, expression of two upregulated mRNAs increased more than five-fold, while expression of 42 downregulated mRNAs decreased more than five-fold (*P* < 0.05, Figure 2D–2F). The top ten differentially expressed mRNAs are listed in Supplementary Table 2. Expression of *COL9A1*, a chondrocyte marker gene, decreased approximately 25-fold in P5 chondrocytes. As was observed for lncRNAs, these results demonstrate that the expression of many mRNAs is altered during human chondrocyte dedifferentiation *in vitro*, and mRNA downregulation was more common than upregulation during this process. These differentially expressed genes might therefore play regulatory roles in dedifferentiation.

GO analysis showed that the biological processes of protein-DNA complex assembly, protein-DNA complex subunit structure, chromatin assembly, and DNA packaging were significantly downregulated in P5 human chondrocytes compared to P1 chondrocytes. Cellular components, such as protein-DNA complexes and chromosomal components, were also downregulated in P5 human chondrocytes. Compared to P1 chondrocytes, protein isodimerization and dimerization activities as well as protein binding molecular functions were significantly downregulated in P5 chondrocytes (*P* < 0.05, Figure 2G). P5 chondrocytes were enriched in various biological processes (including metabolism of nucleoside diphosphate, biosynthesis of DNA, and regulation of protein serine/threonine kinase activity), cellular components (such as neuron soma, neuron components, and dendritic cells), and molecular functions (including ion channel binding, PDZ domain channels, and regulator activity) compared to P1 cells (*P* < 0.05, Figure 2H). The results of GO analysis suggest that differentially expressed genes may lead to dedifferentiation of human chondrocytes *in vitro* by regulating the expression and activity of these biological processes, cellular components, and molecular functions.

Next, we analyzed the signaling pathways that were significantly altered during human chondrocyte dedifferentiation using pathway analysis. Downregulated pathways included DNA replication, nitrogen metabolism, Hippo signaling, and butyrate metabolism (*P* < 0.05, Figure 2I), while upregulated pathways included P53 signaling, purine metabolism, ErbB signaling, HIF1 signaling, and pyrimidine metabolism (*P* < 0.05, Figure 2J). We therefore hypothesized that differentially expressed lncRNAs may directly or indirectly regulate the expression of target genes, thereby altering the activity of downstream signaling pathways and ultimately leading to human chondrocyte dedifferentiation *in vitro*.

### Validation of select lncRNA expression by real-time qPCR

**Figure 3:**
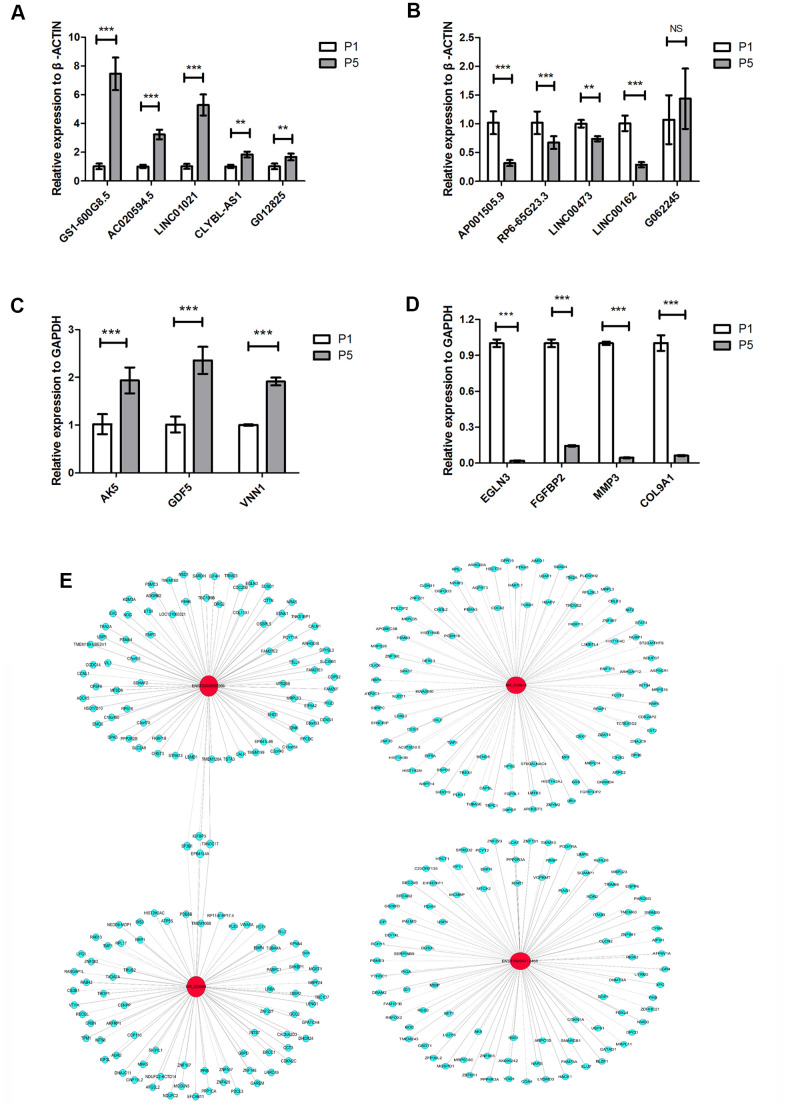
**LncRNA and mRNA expression verified by real-time qPCR.** (**A**) Real-time qPCR results for the upregulated lncRNAs CLYBL-AS1, LINC01021, G012825, GS1-600G8.5, and AC020594.5 in P5 chondrocytes compared to P1 cells from three individual donors. (**B**) Real-time qPCR results for the downregulated lncRNAs AP001505.9, RP6-65G23.3, LINC00473, LINC00162, and G062245 in P5 chondrocytes. (**C**) Real-time qPCR results for the upregulated mRNAs, AK5, GDF5, and VNN1 in P5 chondrocytes. (**D**) Real-time qPCR results for the downregulated mRNAs EGLN3, FGFBP2, MMP3, and COL9A1 in P5 chondrocytes. (**E**) Co-expression network analysis of lncRNAs and mRNAs; red circles represent lncRNAs, blue circles represent mRNAs, solid lines represent positive regulation, and dotted lines represent negative regulation. *GAPDH* and *ACTB* were used as the internal references. Data are represented as means ± standard deviation (n = 3). *P < 0.05, **P < 0.01, ***P < 0.001. NS, not significant.

We selected ten differentially expressed lncRNAs and seven differentially expressed mRNAs for further analysis. The selection criteria for differentially expressed lncRNAs were as follows: (1) high fold-change in expression with smaller P value; (2) raw intensity more than 200; (3) lncRNA length generally less than 2000 bp; (4) no sense-overlap lncRNAs. Ultimately, CLYBL-AS1, LINC01021, G012825, GS1-600G8.5, and AC020594.5, which were upregulated in P5 chondrocytes, and AP001505.9, RP6-65G23.3, LINC00473, LINC00162, and G062245, which were downregulated, were analyzed further. We performed real-time qPCR on P1 and P5 chondrocytes isolated from three donors to quantify the expression of the selected lncRNAs and mRNAs. Except for G062245 (T268788), qPCR results were consistent with microarray analysis results for the selected lncRNAs. LINC01021 (NR_038848) and GS1-600G8.5 (ENST00000412485) showed the largest fold increases in expression among the lncRNAs in the qPCR analysis (*P* < 0.001, Figure 3A), while AP001505.9 (ENST00000569966, LINC00165) and LINC00162 (NR_024089) showed the largest fold decreases (*P* < 0.001, Figure 3B). In addition, qPCR expression results were consistent with microarray analysis results for all selected mRNAs. Specifically, expression of *AK5, GDF5*, and *VNN1* was upregulated, while expression of *EGLN3, FGFBP2, MMP3*, and *COL9A1* was downregulated, during chondrocyte dedifferentiation (*P* < 0.001, Figure 3C, 3D). These results confirmed the accuracy of the microarray analysis results. Next, we examined the gene co-expression networks of AP001505.9, LINC00162, LINC01021, and GS1-600G8.5 to identify potential target genes for future research (Figure 3E). These lncRNAs may promote or inhibit the dedifferentiation of human hyaline chondrocytes by promoting or inhibiting the expression of the associated coding genes.

### LncRNA expression is reversed during chondrocyte redifferentiation

**Figure 4:**
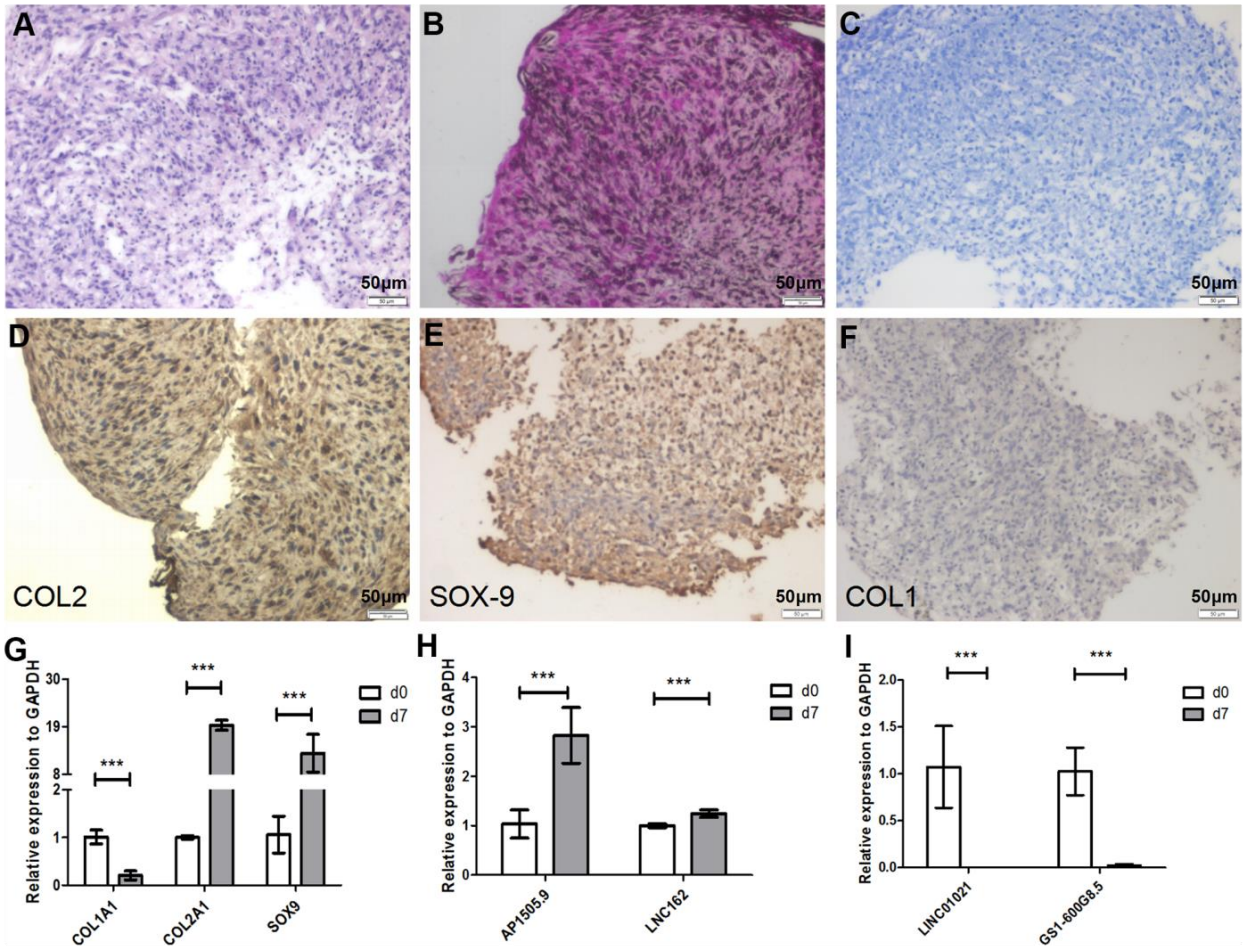
**Seven days of pellet culture induces redifferentiation of P5 chondrocytes.** (**A**) Hematoxylin-eosin (HE) staining results. (**B**) Safranin O staining. (**C**) Alcian staining. (**D**) Immunohistochemistry for COL2. (**E**) Immunohistochemistry for SOX-9. (**F**) Immunohistochemistry for COL1. (**G**) Real-time qPCR for the cartilage-related marker genes *COL1A1*, *COL2A1*, and *SOX*-*9*. (**H**, **I**) Real-time qPCR for the lncRNAs AP001505.9, LINC00162, LINC01021, and GS1-600G8.5. *GAPDH* was used as the internal reference. Data are represented as means ± standard deviation. *P < 0.05, **P < 0.01, ***P < 0.001. Scale bar, 50 μm.

Dedifferentiated chondrocytes can be redifferentiated using pellet culture [21]. To further clarify whether changes in lncRNA expression that occur during chondrocyte redifferentiation can be reversed, P5 chondrocytes were cultured in pellet culture *in vitro* for 7 days. After pellet culture, hematoxylin-eosin (HE) staining indicated that the cells exhibited typical hyaline chondrocyte characteristics (Figure 4A). We then examined the contents of the chondrocyte extracellular matrix using safranin O and Alcian blue staining. Cartilage staining intensity increased significantly in the extracellular matrix after pellet culture (Figure 4B, 4C). To determine whether the cultured chondrocytes were redifferentiated, we analyzed chondrocyte markers at the mRNA and protein levels. COL1A1 mRNA expression was significantly downregulated, while COL2A1 and SOX-9 expression were significantly upregulated, after pellet culture (*P* < 0.001 Figure 4G). At the protein level, COL2 and SOX-9 expression were significantly upregulated, while COL1 expression was significantly downregulated (Figure 4D–4F), after pellet culture. This suggests that dedifferentiated P5 chondrocytes can be redifferentiated using pellet culture.

We then quantified expression of previously selected lncRNAs during redifferentiation. Real-time qPCR analysis showed that AP001505.9 and LINC00162 were upregulated, while LINC01021 and GS1-600G8.5 were downregulated (*P* < 0.001, Figure 4H, 4I), suggesting that changes in lncRNA expression that occurred during chondrocyte dedifferentiation were reversed during chondrocyte redifferentiation.

### Overexpression of AP001505.9 promotes maintenance of the human chondrocyte phenotype and inhibits dedifferentiation

**Figure 5:**
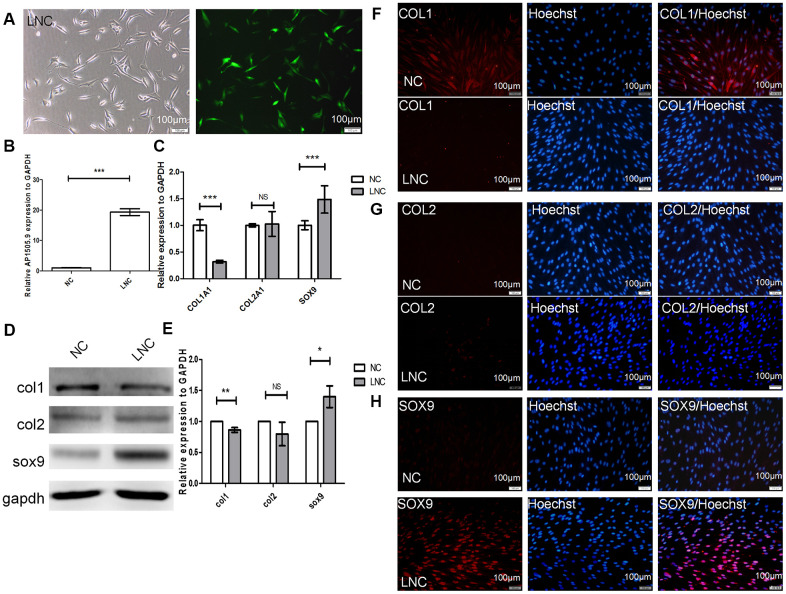
**Transfection of AP001505.9 overexpression lentivirus.** P5 human chondrocytes were inoculated into a 24-well plate at a density of 5 × 10^4^/well. After 24 h, culture medium was replaced with viral transfection solution. Cells were transduced with AP001505.9 overexpression viruses with a multiplicity of infection (MOI) = 75. (**A**) Phase contrast microscopy and fluorescence microscopy; green fluorescence represents successful chondrocyte transfection. LNC represents chondrocytes transfected by AP001505.9 lentivirus. NC represents chondrocytes transfected by negative control lentivirus. (**B**) Real-time qPCR for AP001505.9. (**C**) Real-time qPCR for *COL1A1*, *COL2A1*, and *SOX-*9. (**D**, **E**) Western blotting analysis of COL1, COL2, and SOX-9 levels. (**F**–**H**) Immunofluorescence staining of COL1, COL2, and SOX-9; NC and LNC chondrocytes are stained with anti-COL2, anti-COL1, anti-SOX-9, and Hoechst 33342. *GAPDH* was used as the internal reference. Data are represented as means ± standard deviation. *P < 0.05, **P < 0.01, ***P < 0.001. NS, not significant. Scale bar, 100 μm.

AP001505.9 was downregulated during dedifferentiation and upregulated during redifferentiation. To further analyze the effects of AP001505.9 on the expression of the chondrocyte marker genes *COL1A1, COL2A1,* and *SOX9*, we transfected P5 chondrocytes with lentiviruses that induced overexpression of AP001505.9. Fluorescence microscopy indicated an 80% transfection efficiency (Figure 5A). Real-time qPCR showed that AP001505.9 expression increased approximately 20-fold (*P* < 0.001, Figure 5B), confirming successful lentiviral transfection. We next examined the effects of AP001505.9 overexpression on chondrocyte phenotype and dedifferentiation. The RT-qPCR results indicated that AP001505.9 overexpression significantly upregulated SOX9 expression and downregulated COL1A1 expression (*P* < 0.001); no significant change was observed in COL2A1 expression (*P* > 0.05, Figure 5C). The same expression changes for SOX9 and COL1, as well as the lack of change in COL2, were observed at the protein level (Figure 5D–5H). These results suggest that overexpression of AP001505.9 promoted maintenance of the hyaline chondrocyte phenotype and inhibited dedifferentiation in chondrocytes.

**Figure 6:**
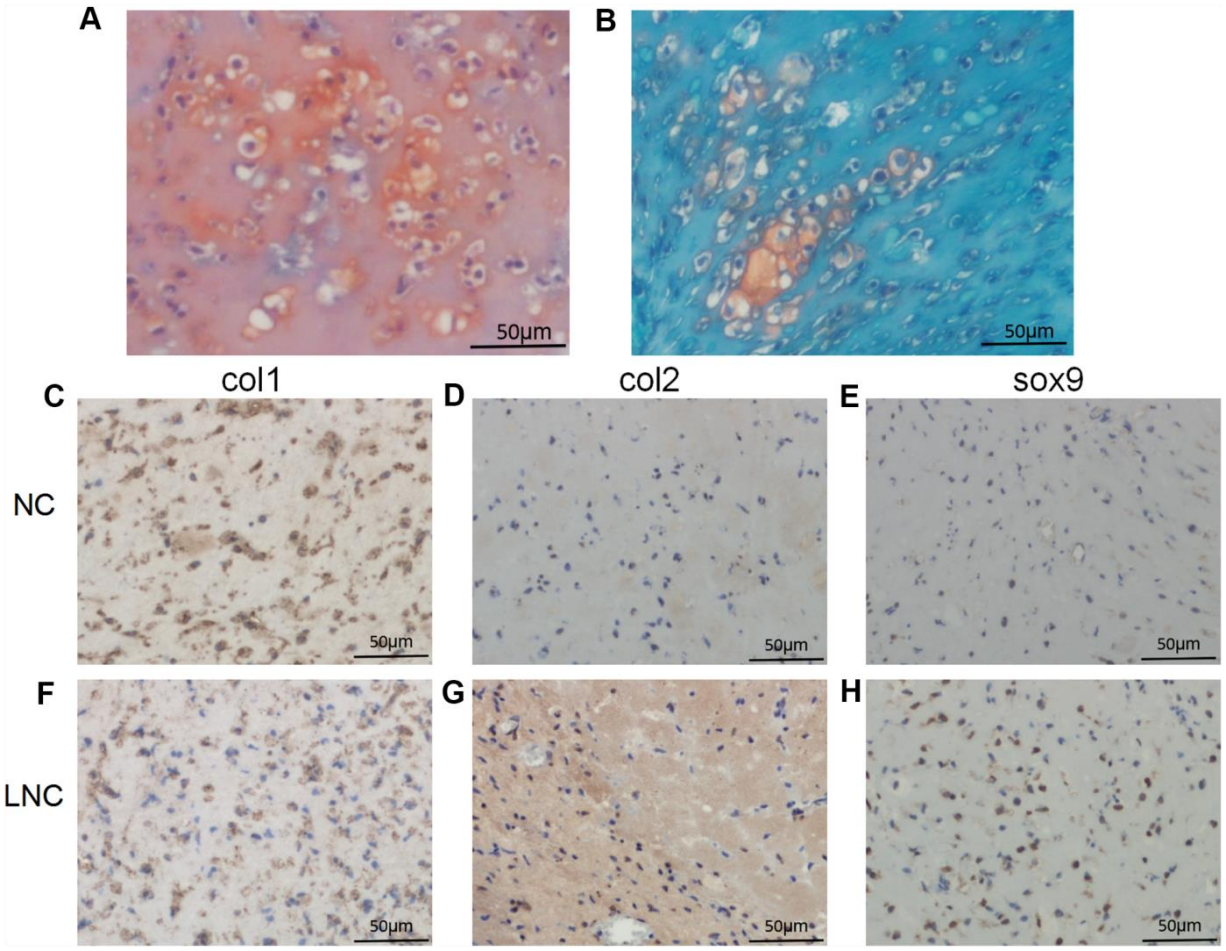
**Histological and immunohistochemical analysis of *in vivo* transplantation.** (**A**, **B**) Safranin O staining. Immunohistochemistry for (**C**, **F**) COL1, (**D**, **G**) COL2, and (**E**, **H**) SOX-9. LNC represents chondrocytes transfected by AP001505.9 lentivirus. NC represents chondrocytes transfected by negative control lentivirus. Scale bar, 50 μm.

To further investigate whether AP001505.9 can maintain human hyaline chondrocyte phenotype and inhibit dedifferentiation *in vivo*, we performed an *in vivo* subcutaneous transplantation experiment in nude mice. Compared to the control group, the pellet formed by transplantation of AP001505.9 overexpression chondrocytes exhibited typical cartilage morphology and increased extracellular matrix collagen content (Figure 6A, 6E). Moreover, COL2 and SOX-9 expression were significantly higher, while COL1 expression was significantly lower, in the AP001505.9 overexpression group than in the control group (Figure 6B–6D, 6F–6H). These results demonstrate that AP001505.9 overexpression also promoted maintenance of the chondrocyte phenotype and inhibited dedifferentiation *in vivo*.

### AP001505.9 inhibits dedifferentiation by regulating SOX-9 expression

**Figure 7:**
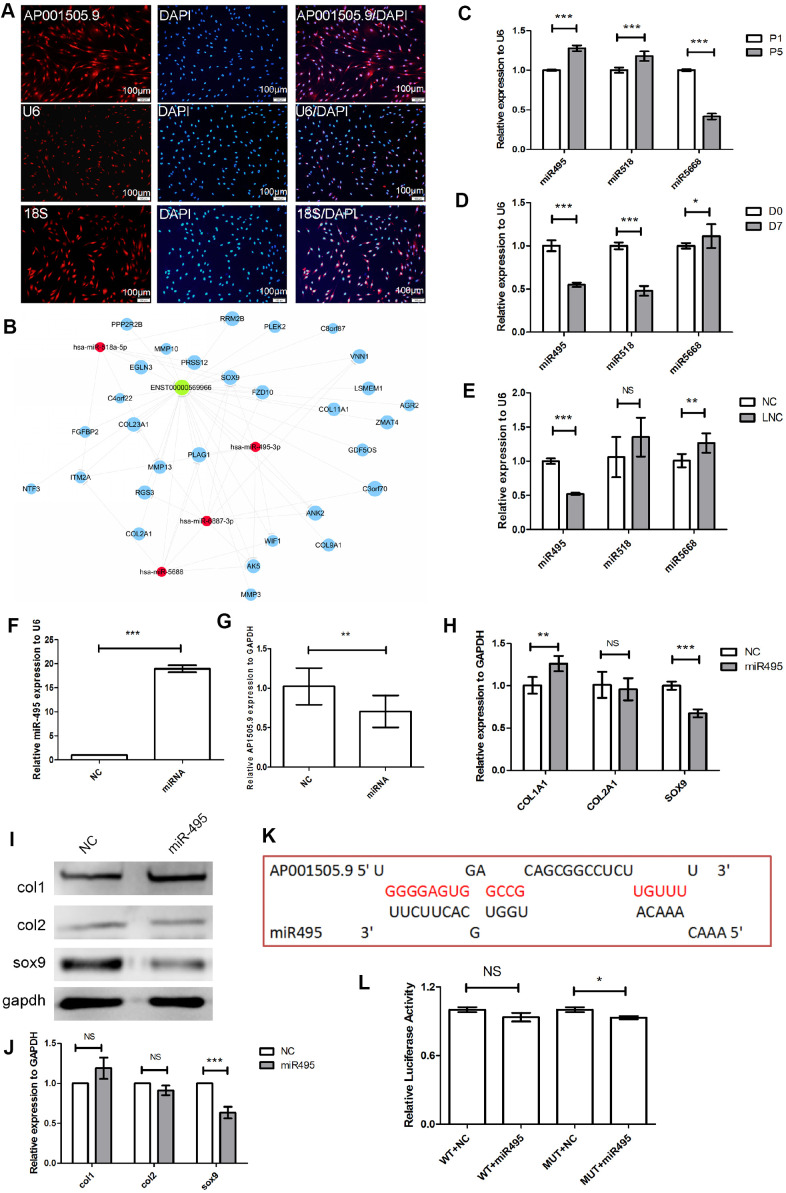
**Mechanism of AP001505.9-dependent inhibition of dedifferentiation.** (**A**) Fluorescence *in*-*situ* hybridization for AP001505.9. U6 and 18S were used as internal reference probes in the nucleus and cytoplasm, respectively. (**B**) Competing endogenous RNA (ceRNA) analysis of AP001505.9; blue circles represent cartilage-related genes; red circles represent miRNAs; green circles represent lncRNAs. (**C**) Real-time qPCR for miRNAs expressed after dedifferentiation. (**D**) Real-time qPCR for miRNAs expressed after redifferentiation. (**E**) Real-time qPCR for miRNAs after AP001505.9 overexpression. (**F**) Real-time qPCR for miR-495. (**G**) Real-time qPCR for AP001505.9 after miR-495 overexpression. (**H**) Real-time qPCR for *COL1A1*, *COL2A1*, and *SOX-9* after miR-495 overexpression. (**I**, **J**) Western blot for COL1, COL2, and SOX-9 after miR-495 overexpression. (**K**) Binding analysis of AP001505.9 and miR-495. (**L**) Double luciferase assay for AP001505.9 and miR-495. LNC represents chondrocytes transfected by AP001505.9 lentivirus. miR495 represents chondrocytes transfected by miR-495 mimic. NC represents chondrocytes transfected by negative control lentivirus or mimic. *GAPDH* was used as the internal reference. Data are represented as means ± standard deviation. *P < 0.05, **P < 0.01, ***P < 0.001. NS, not significant. Scale bar, 100 μm.

To investigate the specific mechanism by which AP001505.9 inhibits hyaline chondrocyte dedifferentiation, we first examined its cellular sublocalization using 18S as the positive control probe for the cytoplasm and U6 as the positive probe for the nucleus. The results showed that AP001505.9 expression was localized to both the nucleus and the cytoplasm (Figure 7A). To explore whether AP001505.9 promoted SOX-9 expression through competitive binding of related miRNAs, we selected genes related to cartilage differentiation, miRNAs, and AP001505.9 for competing endogenous RNA (ceRNA) analysis. Results showed that AP001505.9 may regulate SOX-9 expression by competing with four miRNAs: hsa-miR-495-3p, hsa-miR-518a-5p, hsa-miR-5688, and hsa-miR-6887-3p (Figure 7B). Furthermore, real-time qPCR results showed that miR-495-3p and miR-518a-5p expression were significantly upregulated during dedifferentiation of human chondrocytes, while miR-5688 expression was significantly downregulated (*P* < 0.001, Figure 7C). MiR-495-3p and miR-518a-5p expression were also significantly downregulated after redifferentiation (*P* < 0.001, Figure 7D). Meanwhile, overexpression of AP001505.9 inhibited miR-495-3p expression (*P* < 0.001) and had no significant impact on miR-518a-5p and miR-5688 expression (*P* > 0.05, Figure 7E). These results suggest that AP001505.9 may promote SOX-9 expression by competing with miR-495-3p.

SOX-9 was previously identified as a target of miR-495-3p [34]. We therefore established miR-495 overexpression chondrocytes using miR-495-3p mimics to examine regulation of SOX-9. MiR-495-3p expression increased nearly 20-fold after transfection (*P* < 0.001, Figure 7F). Additionally, AP001505.9 was significantly downregulated (*P* < 0.01, Figure 7G). Moreover, SOX-9 expression was significantly downregulated at both the mRNA and protein levels (*P* < 0.001, Figure 7H–7J). To examine whether AP001505.9 can directly sponge miR-495-3p, we screened AP001505.9 for potential miR-495-3p binding sites (Figure 7K) in a double luciferase experiment. Interestingly, wild-type AP001505.9 vector fluorescence was not significantly downregulated by the miR-495-3p mimic, suggesting the possibility of an indirect co-regulatory mechanism (Figure 7L). AP001505.9 therefore does not serve as a ceRNA of miR-495-3p, and other mechanisms may be responsible for regulating SOX9 expression in this context. Based on these results, we conclude that AP001505.9 overexpression promotes SOX-9 expression and inhibits COL1 expression, thus inhibiting human chondrocyte dedifferentiation.

## DISCUSSION

Articular cartilage injury and degeneration cause significant societal and personal economic burdens [35]. The rise of ACI technology has improved rehabilitation of patients with these injuries and is currently considered the best treatment option [36]. Although various types of stem cells, such as bone marrow mesenchymal stem cells, embryonic stem cells, pluripotent stem cells, and umbilical cord blood stem cells, can be used as seed cells, each method involves inherent challenges, including ethical considerations, differentiation instability, and cancer risk [37]. Human autologous chondrocytes are therefore considered the most suitable seed cells and have been used in ACI. However, their application is limited due to their tendency to dedifferentiate during subculture; this process is characterized by decreases in the hyaline chondrocyte phenotype and increases in the fibrochondrocyte phenotype, conditions that resemble those of OA formation [23].

A popular subject of research in recent years, lncRNAs have been identified as important players in cartilage differentiation and OA pathogenesis [30–32]. In this study, lncRNA expression profiles were characterized during the process of human hyaline chondrocyte dedifferentiation of *in vitro*, and many differentially expressed lncRNAs were identified. Further investigation indicated that AP001505.9 and LINC00162, which were downregulated during dedifferentiation, may inhibit that process in chondrocytes. In contrast, LINC01021 and GS1-600G8.5 were identified as promoters of chondrocyte dedifferentiation. We then employed pellet culture to induce redifferentiation of dedifferentiated chondrocytes. During this process, the changes in expression of these lncRNAs were reversed; specifically, AP001505.9 expression increased nearly three-fold.

In subsequent analyses, we examined the role of AP001505.9 in chondrocyte dedifferentiation as well as the underlying molecular mechanisms. AP001505.9, also known as LINC00165, is an 824-nucleotide intergenic lncRNA located on chromosome 21. Studies have found that LINC00165 is highly expressed in gastric cancer tissues and can promote epithelial-mesenchymal transition in gastric cancer cells, thus promoting their proliferation, migration, and invasion [38]. However, there are no previous reports on the role of LINC00165 in human hyaline chondrocyte dedifferentiation.

Pathway analysis revealed significant changes in expression of signaling pathway members during dedifferentiation. Specifically, DNA replication, nitrogen metabolism, Hippo signaling, and butyrate metabolism pathways were significantly downregulated, while P53 signaling, purine metabolism, ErbB signaling, HIF1 signaling, and pyrimidine metabolism pathways were significantly upregulated. Studies have shown that some of these signaling pathways, such as DNA replication [39], Hippo signaling [40], p53 signaling pathway, [41] and HIF1 pathway [42], are also involved in the development and progression of OA. These results demonstrate that the process of dedifferentiation is similar to that of OA pathogenesis.

LncRNAs can participate in the regulation of gene expression through a variety of mechanisms, such as gene imprinting, chromatin remodeling, and regulation of mRNA precursor splicing, degradation, and translation [25]. However, lncRNAs also act as ceRNAs by sponging target miRNAs, thus promoting the expression of target genes [43, 44]. SOX-9 is an HMG-box (high mobility group box) transcription factor that plays an essential role in chondrocyte development by directing the expression of chondrocyte-specific genes [44]. In fact, SOX-9 can promote the expression of chondrocyte marker genes and inhibit dedifferentiation [45]. Additionally, SOX-9 knockout significantly decreased the expression of chondrocyte marker genes [46]. Many miRNAs, such as miR-145 [47], miR-495 [29], and miR-1247 [48], can also regulate the expression of SOX-9 in chondrocytes, thus promoting the pathogenesis of OA. In our ceRNA analysis, we found that AP001505.9 may regulate SOX-9 expression through four miRNAs: miR-495-3p, miR-518a-5p, miR-5668, and miR-6887. Further examination of these miRNAs revealed that miR-495-3p expression was significantly upregulated during dedifferentiation and downregulated during redifferentiation induced by pellet culture. Meanwhile, AP001505.9 overexpression significantly decreased miR-495-3p expression. However, a luciferase assay indicated that AP001505.9 does not bind directly to miR-495-3p, suggesting that AP001505.9 does not act as a ceRNA by sponging miR-495-3p. The underlying molecular mechanism by which AP001505.9 overexpression promotes SOX-9 expression therefore requires further investigation. Potential alternate mechanisms include gene imprinting, chromatin remodeling, mRNA precursor splicing, mRNA degradation, or translation regulation [25]. In the future, we will investigate whether AP001505.9 can maintain chondrocyte phenotype and inhibit dedifferentiation in animal models of cartilage injury. We will also examine cartilage in OA to investigate the expression profiles of AP001505.9, as the process of dedifferentiation is similar to the process of OA.

In conclusion, this study examined the expression and effects of lncRNAs in human hyaline chondrocyte dedifferentiation *in vitro*. We identified many lncRNAs that were upregulated or downregulated during dedifferentiation and determined that AP001505.9 can inhibit dedifferentiation and promote maintenance of the chondrocyte phenotype by regulating SOX-9. This discovery paves the way for further investigations into mechanisms of dedifferentiation and OA treatment. Additional functional studies of lncRNAs are required to further explore related underlying regulatory mechanisms.

## MATERIALS AND METHODS

### Articular cartilage donors

Human cartilage tissues were harvested from donors immediately after death or trauma (*n* = 6; age = 39–67 years; 3 males, 3 females). All tissues were examined using safranin O staining and graded according to a modified Mankin scale. Tissues with scores < 2 were considered normal hyaline cartilage. Patients or their families provided signed informed consent. This study was approved by the Clinical Ethics Committee of China-Japan Union Hospital of Jilin University (2020032604).

### Passage culture of chondrocytes *in vitro*

Cartilage tissues were cut into small pieces and incubated overnight in Dulbecco’s modified Eagle’s medium (DMEM; HyClone, SH30023.01) containing 1 mg/mL type II collagenase (Sigma, YDM2138) at 37° C and 5% CO_2_. Primary chondrocytes were cultured in DMEM supplemented with 10% fetal bovine serum (FBS; TransGen Biotech Co. Ltd., Beijing, China, FS201-02) and 1% penicillin-streptomycin (Invitrogen, USA, 15140122) at a density of 25,000 cells/cm^2^ at 37° C and 5% CO_2._ The culture medium was changed every two days. Once more than 80% of chondrocytes fused, cells were passaged with 0.25% trypsin-EDTA (Gibco, USA, 25200056). This was repeated until the fifth passage (P5), and P1 and P5 chondrocytes were used in further experiments.

### Real-time quantitative PCR (qPCR)

Total RNA was extracted using TRIzol reagent (Invitrogen, 15596026), according to the manufacturer’s instructions. One-Step gDNA Removal (TransGen) reagent and cDNA Synthesis SuperMix (TransGen, AT311-03) were used for mRNA and lncRNA real-time qPCR according to manufacturer’s instructions. The All-in-One First-Strand cDNA Synthesis Kit (GeneCopoeia, USA, QP014) was used to synthesize cDNA for miRNA real-time qPCR. Real-time qPCR assays were performed using the Applied Biosystems 7300 Plus Real-time PCR System. The ChamQ Universal SYBR qPCR Master Mix (Vazyme, Nanjing, China, Q711) kit was used for mRNA and lncRNA real-time qPCR. Target gene primers (Supplementary Table 3) were purchased from Sangon Biotech Co. Ltd, Shanghai, China. The All-in-One miRNA qRT-PCR Reagent Kit (GeneCopoeia, QP002) was used for real-time qPCR of miRNAs. MiRNA primers are listed in Supplementary Table 4. Reactions were performed according to the manufacturer’s instructions. *GAPDH* (glyceraldehyde-3-phosphate dehydrogenase), *ACTB*, and *U6* served as internal references and relative quantitative statistical analysis was performed using the 2^-ΔΔCt^ method.

### Western blotting

Chondrocyte total protein was extracted using RIPA (radioimmunoprecipitation assay) Lysis Buffer (Beyotime, P0013D) containing 1% phenylmethylsulfonyl fluoride (Beyotime, P1011) as a protease inhibitor. Proteins (10–30 mg) were then separated using 10% PAGE and transferred to polyvinylidene difluoride membranes. After elution, anti-COL2 (Rockland, ab17771), anti-COL1 (Abcam, ab34710), anti-SOX-9 (Millipore, AB5535), anti-β-actin (CMC TAG), and anti-GAPDH (CMC TAG, AT0002) primary antibodies were added and incubated at 4° C overnight. Goat anti-rabbit/anti-mouse IgG (Amersham Pharmacia Biotech Ltd.) secondary antibodies were labeled with horseradish peroxidase (ELISA) and the hypersensitive ECL (enhanced chemiluminescence) kit was used to visualize antibody binding. ImageJ software 1.51j was used to analyze and quantify gray values in electrophoresis images.

### Immunofluorescence

Cells were inoculated into 24-well plates at a density of 5 × 10^4^/well, incubated for 24 h at 5% CO_2_ and 37° C, fixed with 4% paraformaldehyde, perforated with 0.1% Triton X-100, and incubated with 10% goat serum for 1 h. Anti-COL2 (Rockland), anti-COL1 (Abcam), and anti-SOX-9 (Millipore) primary antibodies were added and incubated at 4° C overnight. Goat anti-rabbit/anti-mouse IgG (Amersham Pharmacia Biotech Ltd.) secondary antibodies labeled with horseradish peroxidase (ELISA) were added for 1 h, and Hoechst 33342 (Beyotime) was used to stain the nuclei. Results were observed using an inverted phase contrast fluorescence microscope.

### Microarray analyses

The Arraystar Human LncRNA Microarray (v4.0) was used to detect the expression of 40,173 lncRNAs and 20,730 coding transcripts. Hybridized arrays were washed, fixed, and scanned using the Agilent DNA Microarray Scanner System (part number G2505C). Agilent Feature Extraction software (version 11.0.1.1) was used to analyze the acquired array images. Pathway analysis and GO analysis were used to determine the roles of the differentially expressed mRNAs in the biological pathways or GO terms. Hierarchical clustering and combined analysis were performed using in-house scripts.

### Coding-non-coding gene co-expression (CNC) network

The CNC network was constructed based on correlation analysis between differentially expressed lncRNAs and mRNAs. LncRNAs and mRNAs with Pearson correlation coefficients of 0.99 or greater were selected to construct the network using open-source bioinformatics software Cytoscape v2.8.3 (Institute of Systems Biology in Seattle).

### CeRNA analyses

Potential miRNA targets were predicted using software based on TargetScan and miRanda. A ceRNA network was constructed by merging commonly targeted miRNAs.

### Chondrocyte redifferentiation

P5 chondrocytes were inoculated in a 15 mL centrifuge tube at a density of 2 × 10^5^ cells and cultured with 0.5 mL cartilage induction medium (DMEM High Glucose; TransGen Biotech Co. Ltd., Beijing, China) containing 0.4% sodium pyruvate, 0.1% proline, 0.25% vitamin C, 0.1% TGF-β3, 1% ITS (insulin-transferrin-selenium), 0.2% dexamethasone, and 1% penicillin-streptomycin (Invitrogen, USA, 15140122) at 37° C and 5% CO_2_. The culture medium was replaced every two days, and cultures were terminated on day 7.

### Histological staining

HE staining: After 48 h of fixation, cartilage pellets were embedded in paraffin, sliced, dewaxed using xylene, and washed. Sections were then soaked in hematoxylin solution for 5 min, differentiated in 1% hydrochloric acid-alcohol mixture, dipped in weak aqueous ammonia, stained in eosin dye solution for 15 min, dehydrated using an alcohol gradient, and sealed.

Safranin O staining: After dewaxing and washing, sections were stained in Fast Green solution for 10 min, washed, differentiated in 1% acetic acid solution, stained in safranin O dyeing solution for 10 min, dehydrated using an alcohol gradient, and sealed.

Alcian blue staining: After dewaxing and washing, sections were stained in Alcian blue for 30 min, washed in water for 5 min, stained in nuclear fixing red dye for 10 min, washed, dehydrated with an alcohol gradient, transparentized with xylene, and sealed.

### Immunohistochemistry

After dewaxing and washing, sections were incubated in peroxidase blocking solution, incubated with normal non-immune animal serum, and then incubated with anti-COL2 (Rockland), anti-COL1 (Abcam), and anti-SOX-9 (Millipore) antibodies overnight. After washing, the sections were incubated with goat anti-mouse/rabbit IgG secondary antibodies labeled with biotin for 10 min, washed with PBS, treated with *Streptomyces* antibiotic-peroxidase solution, and incubated for 10 min. Sections were then stained with a DAB (3,3'-diaminobenzidine) reagent kit according to the manufacturer’s instructions. Next, the sections were re-dyed with hematoxylin solution, dehydrated with an alcohol gradient, transparentized with xylene, and sealed with neutral gum. Images were observed and photographed using an inverted microscope (Olympus).

### Lentiviral transfection

P5 human chondrocytes were inoculated into a 24-well plate at a density of 5 × 10^4^/well and cultured in the same culture medium described above. After 24 h, the media was replaced with viral transfection solution (Genechem Co. Ltd, China) and cells were transfected with AP001505.9 overexpression lentivirus (Genechem Co. Ltd, China) at multiplicity of infection (MOI) = 75. Chondrocytes were subcultured once they reached 80% confluency.

### Fluorescent in-situ hybridization

Cells were seeded at 5 × 10^4^/well in 24-well plates, incubated for 24 h, fixed with 4% paraformaldehyde, and permeated with 0.5% Triton X-100. LncRNA Probe Mix (RiboBio, China) and the FISH Kit (RiboBio) were used for detection according to manufacturer’s instructions. U6 and 18S were used as internal references.

### Double luciferase labeling

293T cells in the logarithmic growth phase were seeded at 1.5 × 10^4^/well in 96-well plates and cultured at 37° C for 24 h. The transfection concentration was 50 nM, and the plasmid concentration was 50 ng/well. Three wells were established per experimental group. 48 h after transfection, the culture medium was discarded, and the fluorescence value was determined by adding 1× PBS and 35 μL/well of luciferase substrate followed by gentle mixing for 10 min. The fluorescence value was determined using a fluorescence photometer after adding 30 μL stop reagent and gently mixing for 10 min.

### miR-495 mimic transfection

Cells were inoculated into a 24-well plate at a density of 5 × 10^4^/well. Once they reached a density of 30–50%, cells were transfected with Transfection Reagent (RiboBio) and miR-495 Mimic (RiboBio) according to the manufacturer’s instructions. After 72 h, RNA and protein were extracted for real-time qPCR and western blotting, respectively.

### Subcutaneous transplantation in nude mice

BALB/C A-nu nude mice (male, 4–5 weeks old, about 15 g) were purchased from Beijing HFK BioScience Co. Ltd. All animal experiments were approved by the Animal Experiment Ethics Committee of the Basic Medical College of Jilin University (2019081). Intraperitoneal injection of 0.8% pentobarbital sodium was used for anesthesia. Using 100 μL of porcine fibrin sealant kit (Guangzhou Bioseal Biotech Co. Ltd., China) as the scaffold material, P5 chondrocytes transfected with LV-AP001505.9 (1 × 10^6^) were transplanted into the left dorsal subcutaneous tissue of ten male nude mice. The same number of chondrocytes transfected with negative control (NC)-lentivirus were transplanted into the right dorsal subcutaneous tissue of the same mice. One month later, all mice were sacrificed, and tissue samples were collected for histological and immunohistochemical analysis.

### Statistical analysis

SPSS19.0 software was used for data analysis. Continuous data were analyzed using *t*-tests and count data were analyzed using chi-squared test. Analysis of variance was performed for all data, and Fisher's least significant difference analysis was applied to account for multiple comparisons. In all experiments, data are expressed as mean ± SD (standard deviation), and *P* < 0.05 was considered statistically significant.

### Availability of data and materials

The array data reported in this paper are available at Gene Expression Omnibus under the accession number GSE145817.

## Supplementary Material

Supplementary Tables
