# Supplementary material for: Overexpression of long non-coding RNA AP001505.9 inhibits human hyaline chondrocyte dedifferentiation
Source: Aging (Albany NY). 2021 Apr 4;13(8):11433–54. doi: 10.18632/aging.202833 (PMC8109079; doi:10.18632/aging.202833)
Supplement: Supplementary Tables [file aging-13-202833-s001.pdf]

SUPPLEMENTARY TABLES

Supplementary Table 1. Top 20 differentially expressed lncRNAs in human chondrocyte dedifferentiation *in vitro*.

Seqname	Genesymbol	Fold change	Regulation	P value
ENST00000610087	RP11-53B2.6	13.61615	down	0.008840036
NR_024089	LINC00162	9.0286718	down	0.000474045
T117110	G027615	8.539106	down	0.002149133
ENST00000605537	RP4-555D20.3	8.1710518	down	0.001764169
NR_026860	LINC00473	7.0314198	down	0.007761257
ENST00000525376	AF186192.1	6.6547277	down	0.000534007
T166066	G038554	6.0541931	down	0.042381964
NR_046283	NEBL-AS1	5.1958752	down	0.006541277
NR_027082	SFTA1P	5.0757797	down	0.015806733
ENST00000554032	RP6-65G23.3	4.6948336	down	0.047019677
T009872	G002089	4.6358255	down	0.004060744
NR_033997	RNF144A-AS1	4.4444077	down	0.013727556
ENST00000569966	AP001505.9	4.3928127	down	0.009667888
ENST00000608286	LL22NC03-N14H11.1	4.0253927	down	0.01127986
T117111	G027616	4.0109597	down	0.002496261
NR_036503	PRKCQ-AS1	3.969014	down	0.00934634
T268788	G062245	3.7967111	down	0.011046808
T327661	G076785	3.6682149	down	0.018013603
ENST00000603720	RP11-297J22.1	3.6442772	down	0.029502834
TCONS_00006002	XLOC_002629	3.6380732	down	0.005400053
NR_038848	LINC01021	13.268177	up	0.002749195
uc001szi.3	AK022997	5.5453205	up	0.023157849
NR_120384	CLYBL-AS1	5.0195295	up	0.030403683
T214806	G049673	4.8163804	up	0.040120208
T191664	G044136	4.6401846	up	0.019490651
ENST00000412485	GS1-600G8.5	3.8646113	up	0.013736066
NR_036540	LINC00622	3.2450684	up	0.00102767
ENST00000609674	RP11-563K23.1	3.1289083	up	0.043366377
uc.185-	uc.185	3.1146988	up	0.017589456
ENST00000451982	LINC00969	2.9062816	up	0.043586644
T328268	G076957	2.8993137	up	0.020211392
ENST00000573950	TAPT1-AS1	2.7537015	up	0.001847727
ENST00000608605	RP11-804H8.6	2.7520865	up	0.039475391
uc003ifn.3	AK090904	2.7447524	up	0.004243131
T132722	G031137	2.7103456	up	0.032493573
ENST00000423781	AC004870.4	2.6490488	up	0.013810326
uc.185+	uc.185	2.5668814	up	0.041669705
NR_024006	LINC00950	2.5124995	up	0.015626844
NR_120623	TCERG1L-AS1	2.4902614	up	0.016778347
NR_131012	NEAT1	2.4842201	up	0.007916494

Supplementary Table 2. Significant differentially expressed mRNAs in human chondrocyte dedifferentiation *in vitro*.

Seqname	Genesymbol	Fold change	Regulation	P value
NM_022073	EGLN3	32.531482	down	0.003047488
NM_031950	FGFBP2	28.505518	down	0.007176272
NM_002422	MMP3	25.838385	down	0.007266615
NM_001851	COL9A1	25.715546	down	0.025907389
NM_006408	AGR2	14.594614	down	0.000418139
NM_004867	ITM2A	14.22944	down	0.000910927
NM_002425	MMP10	13.706152	down	0.011459855
NM_016445	PLEK2	13.044514	down	0.001537248
NM_173662	RNF175	12.99444	down	0.004850343
NM_001242668	C8orf87	11.733266	down	0.003816175
NM_152770	C4orf22	8.5786754	up	0.003444654
NM_003619	PRSS12	6.766859	up	0.029568537
NM_174858	AK5	4.5321298	up	0.017562526
NM_004666	VNN1	3.6649314	up	0.000810995
ENST00000374375	GDF5OS	3.6237528	up	0.009969044
NM_001148	ANK2	3.2715659	up	0.000513397
NM_144488	RGS3	3.0256487	up	0.01713328
NM_002655	PLAG1	3.0189914	up	0.039626551
NM_015713	RRM2B	2.9571532	up	0.002338046
NM_182597	LSMEM1	2.9430627	up	7.06605E-06

Supplementary Table 3. Primers used in mRNA and lncRNA real-time qPCR.

Gene name	Sequences of primers
COL1A1	F:5’GTTGCTGCTTGCAGTAACCTT3’<br>R:5’AGGGCCAAGTCCAACTCCTT3’
COL2A1	F:5’TGGACGATCACGAAACC3’<br>R:5’GCTGCGGATGCTCTCAATCT3’
SOX-9	F:5’AGCGAACGCACATCAAGAC3’<br>R:5’CTGTAGGCGATCTGTTGGGG3’
AK5	F:5’GCAGAGCAAATTATGAGC3’<br>R:5’TAGCTGGGAAGCAAACAGT3’
VNN1	F:5’CCGCTAGCACCATGACTACTC3’<br>R:5’GCTCGAGCTACCAACTTAATGA3’
GDF5	F:5’CGATAAGACCGTGTATGAGT3’<br>R:5’CTCGCAGTGGAAAGCCTCGT3’
EGLN3	F:5’CATCAGCTTCCTCCTGTC3’<br>R:5’CCACCATTGCCTTAGACC3’
FGFBP2	F:5’TGGGAACATTGTTGGAAACC3’<br>R:5’GGTTGTCTGTCAGGGAGAGG3’
MMP3	F:5’AGTCTTCCAATCCTACTGTTGCT3’<br>R:5’TCCCCGTCACCTCCAATCC3’
COL9A1	F;5’TCGATGGCTTTGCTGTGCTGGG3’<br>R:5’TGGGTCGCAGGGGGTCACAAT3’
CLYBL-AS1	F:5'ACCAAGAAGCAGGATAGTTAGG3’<br>R:5’ TGCCAGGCTCATTGTCATA3’
LINC01021	F:5' ACTGACCCTTCAATGTGCCC3’<br>R:5’ CATTTCTCAAGCCCCGTGTT3’
AP001505.9	F:5'GCCTCTTGTTTTCCTTCCC3’<br>R:5’ TGCTTCGTGGTGAGACTCCT3’
RP6-65G23.3	F:5'CTCCCCTTTATGAGGACTGC3’<br>R:5’ CTCAGCATGGTTGTAAGCAGTA3’
G012825	F:5'CTTCCTACTCCAGGTAAACCC3’<br>R:5’ TCTCCATCAGCAGTCCAAAC3’
G062245	F:5'CCCAGTAGGAGGATAGTCAAGG3’<br>R:5’ TGTGGTGATGGGACTTCGTG3’
AC020594.5	F:5'CGCCCACTACAGGAAATGAAG3’<br>R:5’ CCACGGGACAAATACTACTCAGA 3’
LINC00473	F:5'AGCCAAAAGGGTTTAGAGTCAG3’<br>R:5’ GAGCAGGTAGGGAAATGATGTT3’
LINC00162	F:5'CGCTGAACTGCCTGGACTTT3’<br>R:5’ TGTGCGGTCTCCTCTTGGGT3’
GS1-600G8.5	F:5'CGGGGAGTCTTGAGAATGGG3’<br>R:5’ TAATCAGGCCGGAGTTGCA3’
GAPDH	F:5’CGCTCTCTGCTCCTCCTGTT3’<br>R:5’ CCATGGTGTCTGAGCGATGT3’
β-Actin	F:5’GTGGCCGAGGACTTTGATTG3’<br>R:5’CCTGTAACAACGCATCTCATATT3’

Supplementary Table 4. Primers used in microRNA real-time qPCR.

microRNA	Source	Identifier
hsa-miR-518a-5p	GeneCopoeia	HmiRQP0579
hsa-miR-495-3p	GeneCopoeia	HmiRQP0537
hsa-miR-5668	GeneCopoeia	HmiRQP2716
snRNA U6	GeneCopoeia	HmiRQP9001
